# Social Support as a Mediator in the Relationship Between Stigma and Mental Health in Adults Living with HIV

**DOI:** 10.3390/ijerph22060935

**Published:** 2025-06-13

**Authors:** Henry Santa-Cruz-Espinoza, Julio Domínguez-Vergara, Natalia Mavila Guzmán-Rodríguez, Janeth Molina-Alvarado, Jennifer Castañeda-Paredes, Gina Chávez-Ventura

**Affiliations:** 1 Escuela de Psicología, Universidad Autónoma del Perú, Lima 15842, Peru; jennifer.castaneda@autonoma.pe; 2 Dirección de Investigación, Universidad Tecnológica del Perú, Lima 15046, Peru; c20928@utp.edu.pe; 3 Escuela de Psicología, Institutos y Centros de Investigación, Universidad César Vallejo, Trujillo 13009, Peru; nguzmanr@ucv.edu.pe (N.M.G.-R.); jmolina@ucvvirtual.edu.pe (J.M.-A.); gchavez@ucv.edu.pe (G.C.-V.)

**Keywords:** HIV seroprevalence, mediation analysis, human health

## Abstract

Exposure to HIV-related stigma and mental health problems have both been reported by HIV-positive individuals. We analyzed the role of social support as a mediator in the relationship between HIV-associated stigma and mental health among adults living with HIV. A total of 303 people aged 18 years and over (M = 40.5; SD = 11.2) with an HIV diagnosis who were selected using a non-probability convenience sampling method in Trujillo, Peru, participated in this study. The Macro PROCESS program for SPSS was used for data analysis. We found that stigma exerts an indirect effect on the mental health among adults living with HIV, mediated through social support (β = −0.05, SE = 0.02; 95% CI [−0.09; −0.02]). However, stigma does not exert a direct effect on mental health (β = −0.08; *p* = 0.21). It is concluded that social support negatively and fully mediates the relationship between HIV-linked stigma and mental health among adults living with HIV. A higher stigma was associated with lower social support, and lower social support negatively affects mental health. For this reason, strengthening social support networks in adults with HIV may have a positive impact on public health.

## 1. Introduction

HIV/AIDS disease continues to be a global health challenge affecting large numbers of people [1] and is considered one of the top ten health threats [2]. There are 39 million people living with HIV worldwide and more than half a million have died of AIDS-related causes [3]. In the Latin American region, the average rate of new diagnoses of HIV infection is 3.6 per 100,000 inhabitants, with homosexual men, men who have sex with men, transgender women and sex workers being more affected [4].

Despite reports of lower mortality and greater access to timely diagnosis, prevention and treatment [3], AIDS continues to generate health and economic impacts [5]. Moreover, the COVID-19 pandemic caused, in recent years, care for people living with HIV to be interrupted and threatened [3]. Health care for this population group was affected at all levels of care [6,7,8,9], including mental health [10]. This represented a risk for this human group that included people who discontinued treatment, new untreated cases and their sexual partners who were unaware of their disease status.

People living with HIV face significant barriers that affect their quality of life [11,12,13,14,15,16] and psychological well-being [17,18,19,20,21]. For this reason, the mental health of people living with HIV is a critical aspect that deserves attention [22], as it can influence treatment adherence [23,24] and health-related decision making [25], such as using condoms and complying with antiretroviral treatment, which prevents the transmission of HIV.

One of the factors that has been shown to have a significant impact on the lives of people living with HIV is the stigma associated with this disease [19,26,27,28,29,30]. HIV stigma is defined as social disapproval characterized by labeling, rejecting and/or discriminating against people only because of their HIV-positive status [31]. From the perspective in which people experience stigma, there are three main forms: (a) enacted stigma, referring to the perception of past or present experiences of prejudice and discrimination; (b) anticipated stigma, which is the degree to which people expect to have future experiences of prejudice and rejection; and (c) internalized stigma, where one adopts negative beliefs and feelings about oneself related to one’s HIV status [32].

HIV-related stigma has four dimensions. The first is linked to experiences of discrimination and rejection when others know about the condition. The second is related to the concern about disclosing one’s HIV status. The third comprises a negative self-image. Finally, the fourth dimension is dominated by concern about the popular opinion about HIV infection [33]. This is corroborated by scientific evidence showing stigma expressed in forms of rejection and discrimination [18,34,35,36,37]. So too, negative internalization of discriminatory and prejudicial experiences can lead to self-stigmatization [38] with profound consequences on the quality of life [39] and mental health of affected individuals [40,41]. Therefore, people living with HIV run the risk of discontinuing their treatment to avoid facing possible discrimination from health professionals; they may also avoid informing their sexual partner of their diagnosis to avoid rejection, which would aggravate the problem by spreading the virus.

To counteract the stigmatizing nature of HIV in people who suffer from it, social support is important [42,43]. This is understood as the perception and belief of feeling loved and appreciated by the social groups to which an individual belongs, such as family, friends and others they consider important in their lives [44]. It plays a relevant role in providing support [45] and contributing to the recovery of physical and emotional health [46,47,48,49]. It is a protective factor in a variety of health contexts [50,51,52], improves self-management, and prevents the emergence of risky behaviors [53].

In the field of mental health, depressive symptoms are more likely to manifest in people living with HIV/AIDS who have low social support than those who have higher levels of support [54,55]. Social support has also been found to be inversely related to suicidal ideation [56], while it has a direct relationship with self-esteem [57]. In addition, social support contributes to the improvement of mental health in gay and HIV-positive men, indirectly promoting their quality of life [58]. In short, the research presented highlights the importance of social support in the well-being of people living with HIV.

Among the studies conducted on social support in people living with HIV, its negative relationship with perceived stigma has been reported [59]. Another study found that both variables are associated with depressive symptoms in pregnant women living with HIV [60]. Also, quality of life was associated with social support and stigma in people living with HIV in India [61]. Although these studies coincide in showing the inverse relationship between stigma and social support in people living with HIV, one study showed an absence of a relationship between the two [62], so the relationship requires clarification.

Regarding the mediating role of social support, it has been able to explain the relationship between assertiveness in coping with difficult experiences and immune responses in people living with HIV in India [63]. Also, it has a mediating effect between HIV self-stigma and depression in men who have sex with men [64]. Another study found social support to be a mediator between disease stigma and medical mistrust because of sexual orientation in gay individuals, bisexual individuals, and men who have sex with men [65]. These findings highlight the mediating role of social support between stigma and some of its clinical or social consequences. However, its influence on the mental health of people living with HIV needs to be explored further.

The current state of previous research has shown: (a) an association between stigma and clinical symptoms denoting poor mental health in people with HIV [40,62,66]; (b) an inverse relationship between social support and clinical symptomatology [54,55,67]; (c) inconclusive findings on the relationship between stigma and social support [59,62]; (d) a mediating role of social support between stigma and other factors that might be linked to mental health [28,63,64,65]. However, there are still gaps in knowledge that merit investigation in people living with HIV, such as: (a) whether there is a direct relationship between stigma and mental health, and not just clinical symptomatology; and (b) whether the indirect effect of stigma on mental health, mediated through social support, could attenuate the impact of the relationship. Therefore, the main objective of this study was to analyze the role of social support as a mediator in the relationship between HIV-associated stigma and the mental health among adults living with HIV.

The theoretical perspective assumed is based on health psychology and takes as a reference a biopsychosocial model that points out not only the presence of biological factors as a determinant of health problems, but also considers relevant the psychological, social and cultural aspects that contribute to the approach to a disease [68]. This model focuses on the person and their biological composition within a personal (personality, experiences, expectations, etc.) and temporal (period of life and illness) context. The person manifests their behavior within a social context where interactions are full of meanings, leading to decision making and evaluation of their quality of life based on their social interactions [69].

The assumed theoretical model explains the presence of psychological problems related to HIV infection that contribute to increased stigmatization and rejection of people living with HIV [70,71]. However, good accompaniment and acceptance by social support networks increase optimism, hope and motivation for better decision making related to their health [17].

The findings of the present study seek to contribute to the identification of effective intervention strategies that improve the quality of life of adults living with HIV by understanding social support as a variable that explains the process of the relationship between stigma and mental health.

## 2. Materials and Methods

### 2.1. Participants and Procedure

The participants in the sample are recipients of a free program of the Ministry of Health, in a public hospital in the city of Trujillo (Peru), in charge of providing free, comprehensive, multidisciplinary treatment to ensure adherence to treatment for patients diagnosed as HIV positive. The program includes specialized professional care, clinical tests (including viral load), multidisciplinary treatment and free follow-up. Professional care also includes addressing the most frequent comorbidities, such as anxiety, depression, personality disorders and dependence on psychoactive substances.

The population of program users who attended the Psychology Clinic in 2024 was represented by 491 patients. The sample composition took into account the recommendations for structural equations [72] using Soper software (2020) [73]. The observed power was estimated with the following parameters for the sample calculation: an effect size d Cohen = 0.2, a desired statistical power of 0.8 and a probability level of 0.05. An upper sample size of 303 participants was reached.

Permission was requested from the hospital authorities for data collection and the participants gave their informed consent. Those who met the following inclusion criteria were evaluated: being over 18 years of age and having been diagnosed with HIV at least one year prior. Failure to sign the informed consent form was considered an exclusion criterion.

The participants were recruited through non-probabilistic convenience sampling since patients who had appointments scheduled for care at the Psychology Office were evaluated. The three questionnaires were applied over a period of 6 months. The evaluation was carried out individually within the Psychology Department of the hospital. Patients received instructions for completing the instruments, verbally and in writing, and then completed the instruments on their own. The evaluating psychologists provided assistance to the participants who only had an elementary school degree to complete the first two items of each psychological test and then they continued on their own.

### 2.2. Measurements

Sociodemographic data sheet. Collected sociodemographic data: age, sex, sexual orientation, employment status, educational level, marital status, history of drug and/or alcohol use, history of disease and time of HIV diagnosis.

HIV stigma (Wrigth et al. [74]). The original scale was composed of 40 items [33] and was later reduced to a 10-item version (e.g., I have been hurt by how people reacted to learning I have HIV) [74]. Each question has 4 Likert-type response options, from 1 = strongly disagree to 4 = strongly agree. The scale was translated into Spanish and validated in the Peruvian context by Ranjit et al. [75]. The translation into Spanish was completed by two Peruvian native speakers and then back-translated into English. Likewise, the translated version was reviewed and discussed through a panel of researchers [75].

The psychometric evidence of the scale was developed through a confirmatory factor analysis, where the 10-item model did not have good fit indices. For this reason, two items that had factor loadings below 0.40 were eliminated. Therefore, the 3-factor model with 8 items showed satisfactory fit indices (χ^2^ = 42.92; gl = 17; *p* < 0.001; RMSEA = 0.06; CFI = 0.98; TLI = 0.95). The reliability was calculated using Cronbach’s alpha coefficient and obtained values above 0.70 in the three factors.

Perception of Social Support Scale ([EPAS]; Vaux et al. [45]). The instrument is composed of 23 items (e.g., My friends respect me), has four response options (strongly agree = 1; agree = 2; disagree = 3 and strongly disagree = 4), and has the dimensions of family, friends and others. The original psychometric properties report acceptable Cronbach’s alpha reliability coefficients (family [α = 0.90]; friends [α = 0.80] and others [α = 0.94]). The scale has been validated in Latin American countries such as Mexico [76] and Chile [77].

Psychometric evidence in the Peruvian context was conducted by Manrique-Millones et al. [78], where by means of the confirmatory factor analysis it established a factor structure of 3 dimensions (BS-χ^2^ (207) = 305.61, CFI = 0.98, RMSEA = 0.05). In addition, the reliability was obtained through the Jöreskog Rho coefficient, presenting acceptable values (0.72 = others, 0.85 = friends and 0.92 = family).

Mental Health Inventory-5 (MHI-5). The instrument was created by Veit and Ware [79] in a general population [80]. It consists of 5 items (e.g., How much of the time, during the last month, have you been a very nervous person?) and uses four response options (never = 1, sometimes = 2, many times = 3 and always = 4). The instrument assesses the presence of well-being and distress.

The psychometric evidence presents a two-dimensional structure with factorial solutions above 0.57. In addition, reliability was assessed through the internal consistency alpha coefficient (α = 0.90). It is applicable in adult and adolescent general populations [81].

### 2.3. Data Analysis

The statistical analysis was performed using the IBM SPSS Statistics version 25 statistical program. At the beginning, the sociodemographic data and descriptive measures of the study variables were analyzed. Subsequently, the reliability was estimated by means of the omega coefficient (ω) for all of the measurement instruments. To consider the reliability acceptable, the value should exceed 0.70. Following this, the bivariate correlation measures among the variables of interest were used. Finally, a mediation model was tested with the Macro PROCESS program for SPSS [82].

For the mediation analysis, the indirect effect of the mediating variable was taken into account [83] and the statistical significance of the mediating variable obtained by bootstrapping 10,000 simulations was estimated. The confidence intervals at 95% were calculated and checked not to be on both sides of zero to be significant for the analysis of the indirect effect [82].

Finally, the correlations of the stigma, social support and mental health with the sociodemographic variables were analyzed, and the effect size was calculated with Spearman correlation statistics for numerical variables, biserial rank correlation for dichotomous and numerical variables, and Epsilon squared effect size for ordinal and numerical variables. For the interpretation of the magnitude of the correlations, the following values were considered: very small (r < 0.12), small (r < 0.24), moderate (r < 0.41) and large (r ≥ 0.41) [84].

## 3. Results

### 3.1. Characteristics of the Participants

The ages of the participants ranged from 20 to 73 years (M = 40.5; SD = 11.2) and the highest proportion of respondents were women (73.6%). All participants were receiving antiretroviral treatment; 90% were following the first-line regimen, i.e., they did not discontinue the first treatment; and 10% were following the second-line regimen, which means that, due to treatment abandonment, they generated resistance to the first treatment and received a higher dosage of medicines.

Although the patients varied in their socioeconomic level, the low level was predominant (50%), followed by medium (40%) and high (10%). Other sociodemographic characteristics are shown in Table 1.

### 3.2. Descriptive Analysis

The findings support a moderate negative correlation of stigma with social support (r = −0.377, *p* < 0.001), a moderate positive correlation of social support with mental health (r = 0.293, *p* < 0.001), and a very small correlation of social support with mental health (r = −0.175, *p* < 0.001). These correlations provide support for the mediation analysis (Table 2).

### 3.3. Mediation Analysis

**Figure 1:**
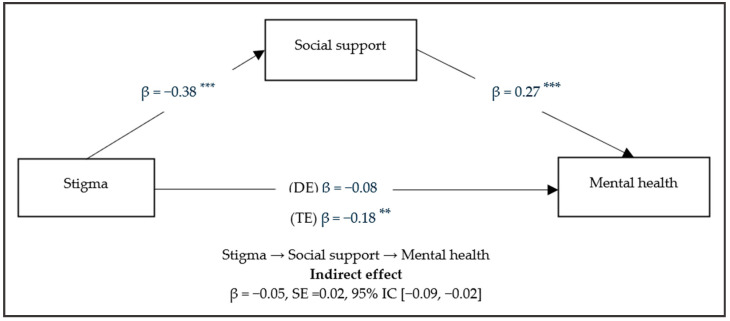
Mediation model of social support between stigma and mental health. DE = direct effect; TE = indirect effect; ** *p* < 0.01; *** *p* < 0.001.

Figure 1 presents the hierarchical linear regression analysis used to explore the mediating role of social support. Stigma shows a negative direct effect on social support (β = −0.38; *p* < 0.001). However, with mental health, no direct effect is evident (β = −0.08; *p* = 0.21). The mediating variable of social support shows a positive direct effect (β = 0.27; *p* < 0.001) on mental health. Finally, the total effect is shown, which refers to the sum of the direct and indirect effects of stigma and mental health, being direct and negative (β = −0.18; *p* = 0.0023).

Table 3 shows the direct and indirect effects of the hypothetical model. It is seen that social support plays a mediating negative role in the relationship of stigma with mental health (β = −0.05, SE = 0.02; 95% CI [−0.09; −0.02]). These results suggest the complete mediation of social support in the relationship of stigma with mental health. Higher stigma was associated with lower social support and lower social support had a negative impact on mental health.

Table 4 shows significant differences according to mental health (rbis = −0.265, *p* < 0.01), obtaining a small effect size and with higher scores in the male sex men than in women. Finally, no correlations were found between stigma, social support and mental health with the other sociodemographic variables.

## 4. Discussion

The purpose of the research was to analyze the role of social support as a mediator in the relationship between HIV-associated stigma and mental health in adults living with HIV. Social support has been found to negatively and completely influence the relationship between HIV-associated stigma and mental health in adults living with HIV. The disapproval, discrimination and social exclusion of people because of their HIV-positive status affects their mental health when they do not have a support network of significant people in their lives.

It is also possible that, due to the stigma associated with an HIV diagnosis, people living with AIDS internalize the stigma and avoid social contact and, therefore, the support provided by significant individuals, which makes them more vulnerable to mental health problems. In other words, social support plays a protective role in mental health by attenuating the impact of discrimination and rejection on people living with HIV.

These findings are similar to that of other previous studies. One of them reported that social support groups reduced the impact of HIV-associated stigma on quality of life in women living with HIV [85]. Similarly, other research found an indirect effect between perceived stigma and depression, partially mediated by social support in men living with HIV who have sex with other men [86]. The referenced antecedents point to social support as a buffer against the impact of stigma in people with HIV.

The beneficial effects of social support in patients living with HIV are reaffirmed [59,60,61] by attenuating the impact of clinical problems [54,55,60]. The importance of a significant support network reduces the likelihood of assumption of risky behaviors [53] that could complicate the health of people living with HIV and any sexual partners they may have.

People just presenting the status of living with HIV experience greater mental health problems [87]. In addition, being part of a sexual minority may be associated with a higher prevalence of mental disorders [88]. In the case of this study, about half of the sample presented a non-heterosexual orientation. Added to this is social stigma, which would be an aggravating factor in people living with HIV, not only because of its relationship with low access to health services and low adherence to treatment [89], but also because it is a barrier to the control of the spread of HIV [90].

Mental health problems are a critical aspect in patients living with HIV that deserve attention [22,86,91], because of their negative influence on treatment adherence [23,24], and on health-related decision making [25]. Therefore, ensuring the well-being of people with HIV would also contribute to preventing the spread of the disease.

The absence of a direct effect between HIV-related stigma and mental health found in this study is in line with a previous study, which found that structural stigma does not directly influence the mental health of LGB people [92]. Other studies, in contrast, reported that HIV-related stigma is associated with manifestations of poor mental health, such as the presence of depression [40,41,86].

These findings support the merits of social support and elucidate how HIV-related stigma has an indirect effect on the mental health of patients with HIV-positive status; this research, however, contains limitations. Having worked with a non-probabilistic sample obtained from hospital contexts affects the external validity of the study. However, since the participants were part of a vulnerable population and psychological constructs that could generate susceptibility were measured, it was necessary that the assessments be applied during psychological counseling in order to maximize well-being and minimize the risks derived from the assessment. To reduce bias, we worked with a large sample that included participants with varied characteristics with respect to educational level, sex and type of treatment.

Another limitation is the use of self-report instruments because they may contain some social desirability bias. However, this type of measurement is relevant because the tests used measure perceptions, beliefs and behaviors. To avoid bias, the identity of the participants, who agreed to participate in the study, was kept anonymous. In addition, the tests used showed adequate psychometric evidence of validity and reliability.

A further limitation is the cross-sectional design, which contains causal inferences that are limited by the impossibility of observing the behavior of the variables over time. A longitudinal design was not applied because, being a vulnerable population, it was essential to guarantee the anonymity of the participants, which prevented locating those evaluated at another time.

Other limitations of the study include the failure to consider the confounding variable of treatment adherence and the possible effect modifier of sexual orientation in the analyses. In the first case, although adherence information was not collected, all participants were receiving treatment at the time of the evaluation. Therefore, in the absence of variability in adherence, it was not possible to analyze this information. In the second case, the small number of cases for each type of sexual orientation did not make it possible to accurately analyze this aspect. However, future studies could include as a moderating variable being part of the LGBTQ+ community, because being part of it is associated with a higher prevalence of mental disorders [87].

In light of the limitations mentioned above, we consider the findings of this study to be robust and important, having used instruments with adequate psychometric quality and because this study is based on previous research and assumed theories. In practice, this study provides evidence for the need to integrate social support as a key factor in interventions for adults living with HIV. Promoting the development of emotional and community support programs can significantly strengthen the well-being of people living with HIV. In addition, the results suggest that health professionals should incorporate strategies to improve patients’ support networks, contributing to the reduction of stigma and promoting mental health in this vulnerable group.

As for future lines of research, it would be relevant to delve deeper into the different forms of social support and their differential impact, considering the quality of the support received. Also, the effectiveness of specific interventions focused on social support to reduce stigma should be explored. Finally, longitudinal studies could provide more information on the long-term effects of social support on the stigma and mental health of people living with HIV.

## 5. Conclusions

The problem of social stigma associated with HIV raises the need for interventions [93,94] to preserve the mental health of those living with HIV and prevent the occurrence of mental health disorders and the consequences they generate in the spread of the disease. In addition, it is necessary that, within the prevention and intervention programs for this population, the social support network available to them should be strengthened or self-help groups should be formed.

## Figures and Tables

**Table 1:** Sociodemographic data of the sample.

Characteristics	(*n* = 303)
Sex (%)	
Woman	223 (73.6%)
Man	80 (26.4%)
Sexual orientation (%)	
Heterosexual	163 (53.8%)
Homosexual	108 (35.6%)
Bisexual	26 (8.6%)
Other	6 (2.0%)
Age (M ± SD)	40.55 ± 11.17
Educational level (%)	
Primary school	32 (10.6%)
Secondary school	131 (43.2%)
Higher technical	57 (18.8%)
Higher education	83 (27.4%)
Marital status (%)	
Single	190 (62.7%)
Married/live-in partner	95 (31.4%)
Divorced/separated	18 (5.9%)
Employment status (%)	
Employed	231 (76.2%)
Unemployed	57 (18.8%)
Student	14 (4.6%)
Retired	1 (0.3%)
Time of diagnosis (M ± SD)	81.8 ± 129.34

**Table 2:** Descriptive statistics and correlation analysis among variables.

Variable	M	SD	Min	Max	1	2
1. Stigma	21.23	5.43	10	58	-	
2. Social support	48.03	7.12	18	91	−0.377 **	-
3. Mental health	15.69	2.87	6	20	−0.175 **	0.293 **

Note: M = mean, SD = standard deviation, Min = minimum value, Max = maximum value, ** *p* < 0.01.

**Table 3:** Direct and indirect effects of the stigma model and mental health mediated by social support.

Model Routes	Estimated Effect	SE	95% CI	*p*
Direct effects				
Stigma → Mental health	−0.08	0.03	−0.10, 0.02	0.2094
Stigma → Social support	−0.38	0.02	−0.63, −0.36	<0.001 ***
Social → Mental health	0.27	0.09	0.06, 0.15	<0.001 ***
Indirect effect				
Stigma → Social support → Mental health	−0.05	0.02	−0.09, −0.02	<0.001 ***
Total effect	−0.18	0.03	−0.15, −0.03	0.0023 ***

Note: EE = standard error, 95% CI = 95% confidence interval of 1000 bootstraps, *** *p* < 0.001.

**Table 4:** Stigma, social support and mental health according to sociodemographic variables.

Variables	Stigma	Social Support	Mental Health
Sex	0.073 ^b^	0.003 ^b^	−0.265 ^b^ **
Age	0.011 ^a^	−0.055 ^a^	−0.025 ^a^
Orientation	0.001 ^c^	0.031 ^c^	0.003 ^c^
Educational level	0.047 ^c^	0.072 ^c^	0.009 ^c^
Marital status	0.003 ^c^	0.000 ^c^	0.004 ^c^
Employment status	0.025 ^c^	0.022 ^c^	0.024 ^c^
Time of diagnosis	0.021 ^a^	−0.036 ^a^	0.002 ^a^

Note: ^a^ Spearman correlation, ^b^ Rank-biserial correlation, ^c^ Epsilon squared effect size. ** *p* < 0.01.

## Data Availability

The database is available on the Open Science Framework with registration number 8hw2a: https://osf.io/8hw2a/?view_only=2123dce718164e03ba18b2d6ad23a9c3 (accessed on 20 April 2025).
